# Factors related to the mortality risk of severe hand, foot, and mouth diseases (HFMD): a 5-year hospital-based survey in Guangxi, Southern China

**DOI:** 10.1186/s12879-023-08109-y

**Published:** 2023-03-08

**Authors:** Yuanjun Peng, Weitao He, Zhigang Zheng, Peijiang Pan, Yu Ju, Zhenwei Lu, Yanyan Liao, Hailong Wang, Chao Zhang, Jing Wang, Lina Jiang, Hao Liang, Minmei Chen, Li Ye

**Affiliations:** 1 Guangxi Key Laboratory of AIDS Prevention and Treatment, School of Public Health, Guangxi Medical University, Nanning, 530021 Guangxi China; 2 Institute of Acute Infectious Disease Prevention and Control, Guangxi Zhuang Autonomous Region Center for Disease Prevention and Control, Nanning, 530028 China; 3 China (Guangxi)-ASEAN Joint Laboratory of Emerging Infectious Diseases, Life Science Institute, Guangxi Medical University, Nanning, 530021 Guangxi China; 4 Department of Epidemiology, School of Public Health and Management, Guangxi University of Chinese Medicine, Nanning, 530200 Guangxi China

**Keywords:** Hand, foot and mouth disease, Severe cases, Outcome, Influencing factors, EV-A71 vaccination

## Abstract

**Background:**

To understand the factors influencing clinical outcomes of severe hand, foot, and mouth diseases (HFMD), and to provide scientific evidence for reducing the mortality risk of severe HFMD.

**Methods:**

From 2014 to 2018, children diagnosed with severe HFMD cases in Guangxi, China, were enrolled in this hospital-based study. The epidemiological data obtained through face-to-face interviews with the parents and guardians. Univariate and multivariate logistics regression models were used to analyze the factors influencing the clinical outcomes of severe HFMD. The impact of the EV-A71 vaccination on inpatient mortality was analyzed by a comparison approach.

**Results:**

A total of 1565 severe HFMD cases were enrolled in this survey, including 1474 (94.19%) survival cases and 91 (5.81%) death cases. The multivariate logistic analysis demonstrated that HFMD history of playmates in the last three months, first visit to the village hospital, time from the first visit to admission less than two days, no correct diagnosis for HFMD at the first visit, and having no rash symptoms were the independent risk factors for severe HFMD cases (all *p* < 0.05). While EV-A71 vaccination was a protective factor (*p* < 0.05). The EV-A71 vaccination group versus the non-vaccination group showed 2.23% of death in the vaccination group and 7.24% of death in the non-vaccination group. The EV-A71 vaccination protected 70.80% of the death of severe HFMD cases, with an effective index of 4.79.

**Conclusions:**

The mortality risk of severe HFMD in Guangxi was related to playmates had HFMD history in last 3 months, hospital grade, EV-A71 vaccination, patients visit hospital previously, and rash symptom. EV-A71 vaccination can significantly reduce mortality among severe HFMD. The findings are of great significance for the effective prevention and control of HFMD in Guangxi, southern China.

**Supplementary Information:**

The online version contains supplementary material available at 10.1186/s12879-023-08109-y.

## Background

Hand, foot, and mouth disease (HFMD) is an acute contagious disease caused by human enteroviruses (EVs), which mostly affects children under five years old. The transmission of HFMD is mainly through direct contact with saliva, faces, vesicular fluid, respiratory droplets of the infected individual, or indirect contact with contaminated objects. Generally, HFMD is a self-limited disease. However, some patients may also develop neurologic complications such as neurogenic pulmonary edema, aseptic meningitis, acute flaccid paralysis, and encephalitis, and even die [1]. Guangxi Zhuang Autonomous Region, located in Southern China, is one of the worst-hit provinces for HFMD. The average morbidity and mortality have consistently ranked first among all provinces in mainland China over the decade. There were about 9,000 severe HFMD cases in Guangxi from 2012 to 2015 [3]. In recent years, the severe cases and fatal cases were on the rise, hundreds of cases with central nervous system complications were emerged, resulting in nearly 100 deaths. This poses a serious public health challenge for local health systems [2, 3]. In addition to a higher risk of death, severe HFMD patients have sequelae such as dysfunctional aerodigestive tract, neurological sequelae, delayed neurodevelopment, impaired cognition even after treatment [4]. The sequelae can greatly reduce the life quality of the patients, increase the burden on families, and cause over-expenditure on the social economy. Enterovirus A71 (EV-A71) was the dominant etiological agent of severe HFMD in Southern China.

Due to the excessive economic and disease burden caused by Enterovirus A71 and no specific drugs to cure this disease in the clinic, the development of EV-A71 vaccines is of great importance for China. Three different inactivated EV-A71 vaccines were approved for the market after the completion of clinical trials by the China Food and Drug Administration, Chinese medical discipline Institute of Medical Biology (Kunming) (licensed for the market in 2015), Beijing Sinovac Biology Products Co., LTD. (Beijing Sinovac) (licensed for the market in 2015) and Wuhan institute of biological products CO., LTD (Wuhan) (licensed for the market in 2016). The EV-A71 inactivated vaccine was developed based on Vero-cell without aluminum hydroxide, and the vaccine strain is generated from a C4 genotype strain (GenBank: GU198367.1). And the Sinovac EV-A71 vaccine has shown immunogenicity, safety, effectiveness, and immune persistence in children older than 6 months [5–7]. Further, the efficacy of over 95% against EV-A71-related HFMD was reported in some studies [8, 9].

Since 2016, Guangxi has launched a universal vaccination campaign against EV-A71 for children under 5 years old. As of December 31, 2019, EV-A71 vaccination cumulative coverage in Guangxi was 32.8%, and 27.3% of children under five years of age had completed the 2-dose vaccination, and the proportion of HFMD cases aged 0–12 months decreased from 23.0% to 15.3% between 2013–2015 and 2017–2019 [10].To the best of our knowledge, few studies have assessed the impact and effectiveness of the vaccination in reducing mortality of HFMD hospitalized patients in real-world settings. Therefore, the aim of this study was to analyze the influencing factors of clinical outcomes of severe HFMD cases in southern China, and to further evaluate the effect of the monovalent inactivated EV-A71 vaccination as a protective factor in reducing mortality.

## Methods

### Study design and study population

A hospital-based epidemiological survey was conducted in Guangxi, a province in southern China where HFMD is prevalent. Cases of severe HFMD from 2014 to 2018 were collected from Guangxi Zhuang Autonomous Region Center for Disease Prevention and Control (CDC) system. The definition of severe HFMD was referred to the “diagnosis and treatment guidelines for HFMD” (2010)” [11], and the diagnosis criteria are as follow: (1) frequent convulsions, coma and cerebral hernia; (2) breathing difficulties, cyanosis, bloody frothy sputum and pulmonary rales; and (3) shock and circulatory insufficiency. In our study, subjects were included if: (1) Severe HFMD cases: clinical severity was defined as the patient experienced any neurological complications (aseptic meningitis, encephalitis, encephalomyelitis, acute flaccid paralysis, or autonomic nervous system dysregulation) and/or cardiopulmonary complications (pulmonary edema, pulmonary hemorrhage, or cardiorespiratory failure) and/or circulatory system symptoms (pale face, cold limbs, fingers (toes) cyanosis, cold sweat, et al.), Severe HFMD cases were classified if the patients experienced any symptoms belonging to the clinical severity, others were categorized as mild cases [11, 12]. (2) Patient’s parents approved of participation; (3) Individuals with completed investigation data. Subjects were excluded if: (1) The neurological dysfunction was caused by non-HFMD; (2) Patients with incomplete investigation data. All participants understood the purpose of the study and signed the informed consent forms. An investigation was performed following the relevant guidelines and regulations; cases of severe HFMD from 2014 to 2018 were collected from Guangxi Zhuang Autonomous Region Center for Disease Prevention and Control (CDC) system, the investigation was done by the staff of the CDC.

The sample size can be calculated by the following formula: $\mathrm{n}= \frac{{Z}_{1-\alpha /2}^{2}*\pi (1-\pi )}{{\delta }^{2}}$. The annual proportion of severe HFMD diseases was set at about 20% [3, 10], then we calculated the sample size using the PASS software.

### Data collection

Detailed clinical data of the severe HFMD patients were collected by reviewing the medical records. Socio-demographic characteristics of the patients were collected via face-to-face interviews with patients and guardians using a structured questionnaire (Additional file 2) [13]. The collected information included demographic characteristics (gender, age, EV-A71 vaccination, the patient was hospitalized for other reasons previously, HFMD history of patient’s daily playmates prior to the onset of the current illness, etc.), disease characteristics, diagnosis, and treatment (date of the illness onset, first diagnosis and admission, severe HFMD diagnosis, clinical severity at admission, hospital grade of initial and severe HFMD diagnosis, rash symptoms, etc.). Questionnaire validation: The structural questionnaire we used to collect data was developed by China CDC. It was recommended to apply to invest the severity hospitalization HFMD cases in mainland China through a National Guideline [13]. The local health authority approved the utilization of the questionnaire.

### Definitions

1. Vaccine protection against death. The percentage of death among vaccinated patients was protected compared with that among unvaccinated patients. The following formula was used to calculate the vaccine protection against death:
  $$\mathrm{Protective \, Effectiveness }\left(\mathrm{PE}\right)= \left(1-\mathrm{ odds \, ratio}\right)*100\mathrm{\%}$$
2. Index of effectiveness against death. It is defined as the number of death in the unvaccinated group divided by the number of deaths in the vaccinated group.

### Statistical analyses

*Statistical sofrware*: All statistical analyses were performed using R software (version 4.04) and IBM SPSS Statistics (version 26).

*Regression model*: Univariate and multivariate logistics regression models were used to analyze the factors influencing the clinical outcomes of severe HFMD. The “Forward” method was used to select the factors into the logistic regression model. The influencing factors were first analyzed by the univariate analysis, then the variables with differences (*p* < 0.1) were included in the multivariate logistic regression model. Variables with significant differences (p ≤ 0.05) are considered in the final interpretation. All the comparisons were two-sided, and the p-value of < 0.05 was considered significant. The OR and 95%CI were compared between the survival group and the death groups.

## Results

### Demographics characteristics

As shown in Table 1, a total of 1565 patients with severe HFMD were enrolled in this study, including 998 males (63.77%) and 567 females (36.23%), with a male-to-female ratio of 1.76:1. The median age of the patients was 1.83 years old (0–11.69 years). Severe HFMD occurred mainly in children under 3 years old (80.57%). There were 1288 severe HFMD cases in rural areas, accounting for 82.30%, and the ratio of rural to urban was 4.75:1. Among all 1565 severe HFMD cases, 91 cases died (5.81%, 91/1565). Univariate analysis indicated that, between the survival group and death group, there was no significant difference in the distribution of gender, age, area, registered residence, group classification, etc. (Table 1) (all *p* > 0.05). While there was a significant difference (*p* < 0.05) in EV-A71 vaccination, visiting hospital previously, HFMD history of playmates in the last 3 months, hospital grade of first visit, correct diagnosis at first visit, time interval from first visit to diagnosis of severe HFMD, clinical severity at admission and rash symptoms (Table 1).

**Table 1:** Epidemiological characteristics of severe HFMD cases in Guangxi, 2014–2018, grouped by survival and death

Characteristics	Total n	Survival n (%)	Death n (%)	χ^2^	*p*
Gender					
Male	998	948 (94.99)	50 (5.01)	3.26	0.07
Female	567	526 (92.77)	41 (7.23)		
Age (years)					
0–1 years	160	144 (90.00)	16 (10.00)	7.36	0.06
1–2 years	687	656 (95.49)	31 (4.51)		
2–3 years	414	389 (93.96)	25 (6.04)		
3 years or older	298	280 (93.96)	18 (6.04)		
Area					
Urban	271	256 (94.46)	15 (5.54)		
Rural	1288	1213 (94.18)	75 (5.82)	0.03	0.85
Registered residence					
Long-term	1527	1441 (94.37)	86 (5.63)		
Migrant	30	27 (90.00)	3 (10.00)	1.04	0.31
Group classification					
Kindergarten children	204	193 (94.61)	11 (5.39)	0.34	0.89
Scattered children	1341	1263 (94.18)	78 (5.82)		
School student	15	14 (93.33)	1 (6.67)		
No. of children 0–5 years old in the family					
≤ 1	678	638 (94.10)	40 (5.90)		
2–3	555	525 (94.59)	30 (5.41)		
≥ 4	125	120 (96.00)	5 (4.00)	0.76	0.69
Milk feeding way					
Breast milk	1 151	1 090 (94.70)	61 (5.30)	0.48	0.79
Milk powder	84	81 (96.43)	3 (3.57)		
Mix feeding	296	281 (94.93)	15 (5.07)		
EV-A71 vaccination					
Yes	628	614 (97.77)	14 (2.23)		
No	925	858 (92.76)	67 (7.24)	19.02	< 0.01
Visiting hospital previously					
Yes	38	33 (86.84)	5 (13.16)		
No	1514	1430 (94.45)	84 (5.55)	3.97	0.04
HFMD history of playmates in the last 3 months					
Yes	231	208 (90.04)	23 (9.96)		
No	1318	1253 (95.07)	65 (4.93)	9.26	< 0.01
History of chicken pox, eczema, etc. in the last month					
Yes	115	112 (97.39)	3 (2.61)		
No	1433	1349 (94.14)	84 (5.86)	2.12	0.14
Time interval from disease onset to first visit to hospital					
≤ 1 day	1321	1245 (94.27)	76 (5.75)	0.81	0.67
1–2 days	134	127 (94.78)	7 (5.22)		
≥ 3 days	103	95 (92.23)	8 (7.77)		
Hospital grade of first visit					
Village	429	390 (90.91)	39 (9.09)	18.44	< 0.01
Township	211	195 (92.42)	16 (7.58)		
County	573	546 (95.29)	27 (4.71)		
City	341	333 (97.65)	8 (2.35)		
Correct diagnosis at first visit					
Yes	1066	1024 (96.06)	42 (3.94)		
No	490	442 (90.20)	48 (9.80)	21.124	< 0.01
Time interval from first visit to diagnosis of severe HFMD					
≤ 1 day	740	691 (93.38)	49 (6.62)	6.91	0.03
1–2 days	361	336 (93.07)	25 (6.93)		
≥ 3 days	452	437 (96.68)	15 (3.32)		
Hospital grade of severe HFMD diagnosis					
County grade or below	607	566	41	1.95	0.16
City grade	947	899	48		
Time interval from first visit to admission					
≤ 1 day	882	829 (93.99)	53 (6.01)	0.95	0.62
1–2 days	328	307 (93.60)	21 (6.40)		
≥ 3 days	336	320 (95.24)	16 (4.76)		
Clinical severity at admission					
Mild	316	300 (94.94)	16 (5.06)		
Severe	1137	1094 (96.22)	43 (3.79)		
Critical	92	61 (66.30)	31 (33.70)	139.25	< 0.01
Fever					
Yes	1514	1428 (94.32)	86 (5.68)	0.25	0.62
No	47	43 (91.49)	4 (8.51)		
Rash symptoms					
Yes	1517	1434 (94.53)	83 (5.47)	13.27	< 0.01
No	31	24 (77.42)	7 (22.58)		

### Multivariate logistic analysis

To further determine the factors influencing the clinical outcomes of severe HFMD, multivariate logistics regression analysis was performed, and the variables with differences (*p* < 0.1) in univariate analysis (Table 1) were included in the multivariate logistic regression model. The status (survival and death) at the end of the follow-up was set as the outcome variable (survival = 0, death = 1). Among the independence variables, we set female, no HFMD history of playmates in last 3 months, EV-A71 vaccination, no visiting hospital previously, first visit to the city hospital, time interval from first visit to diagnosis of severe HFMD ≥ 3 days, correct diagnosis at first visit, having rash symptoms as the reference, respectively, to calculate internal OR values (Table 2). The multivariate logistics regression analysis showed that playmates had HFMD history in last three months (OR: 2.30, 95%CI: 1.31–4.03), no EV-A71 vaccination (OR: 4.98, 95%CI: 2.63–9.43), patients visit hospital previously (OR: 4.23, 95%CI: 1.45–12.33), the first visit to village hospital (OR: 6.67, 95%CI: 2.35–18.94), township(OR: 4.28, 95%CI:1.45–12.61), and county(OR:3.05, 95%CI:1.18–7.85), no correct diagnosis for HFMD at the first visit(OR:3.05, 95%CI:1.18–7.85), the time interval from the first visit to diagnosis of severe HFMD ≤ 1 day (OR: 6.37, 95%CI: 2.74–14.80) or 1–2 days (OR: 3.48, 95%CI: 1.78–6.84), having no rash symptoms (OR: 4.60, 95%CI: 1.54–13.67) were the promoting factors for death risk among severe HFMD cases (Table 2).

**Table 2:** Multivariate logistic analysis of potential risk factors for death in severe HFMD patients

Characteristics	Total(n)	β	SE	Wald χ^2^	*p*	OR	95% CI
HFMD history of playmates in last 3 months							
Yes	224	0.83	0.29	8.40	< 0.01	2.30	1.31–4.03
No	1290					1	
EV-A71 vaccination							
Yes	616					1	
No	898	1.61	0.33	24.26	< 0.01	4.98	2.63–9.43
Visiting hospital previously							
Yes	37	1.44	0.55	6.95	< 0.01	4.22	1.45–12.33
No	11,477					1	
Hospital grade of first visit							
Village	419	1.90	0.53	12.71	< 0.01	6.67	2.35–18.94
Township	204	1.45	0.55	6.96	< 0.01	4.28	1.45–12.61
County	559	1.11	0.48	5.33	0.02	3.05	1.18–7.85
City	332					1	
Correct diagnosis at first visit							
Yes	1039					1	
No	475	0.82	0.30	7.35	< 0.01	3.05	1.18–7.85
Time interval from first visit to diagnosis of severe HFMD							
≤ 1 day	372	1.85	0.43	18.52	< 0.01	6.37	2.74–14.80
1–2 days	703	1.25	0.34	13.17	< 0.01	3.49	1.78–6.84
≥ 3 days	439					1	
Rash symptoms							
Yes	1485					1	
No	29	1.53	0.56	7.52	< 0.01	4.60	1.54–13.67

*OR* odds ratio, *CI* confidence interval, *HFMD* hand, foot and mouth disease, *EV-A71* enterovirus A71

### Analysis of impact of EV-A71 vaccination on death in severe HFMD patients

To further investigate the impact of inactivated monovalent EV-A71 vaccination on death in severe HFMD patients, we described the epidemiological characteristics grouped by vaccination or not, and there were 628 patients and 925patients in the vaccinated and unvaccinated group, respectively. The results showed that there was a significant difference (p < 0.05) between the vaccinated and unvaccinated group in number of children 0–5 years old in the family, milk feeding way, hospital level of the first visit, severe diagnosis hospital level, the time from the first visit to admission (Additional file 1). The analysis indicated that 2.23% of the patients died in the vaccinated group and 7.24% in the unvaccinated group (p < 0.01). The inactivated monovalent EV-A71 vaccine can protect 70.80% of death among severe HFMD cases, and the effectiveness index against death was 4.79 (Table 3).

**Table 3:** Analysis of protective effect of EV-A71 vaccination on death of severe HFMD cases

Outcome	EV-A71 vaccination		Protective effectiveness (%)	Effect index	χ^2^	OR	*p*
	Yes n (%)	No n (%)					
Death	14 (2.23)	67 (7.24)	70.80	4.79	19.02	0.29	< 0.01
Survival	614 (97.77)	858 (92.76)					

## Discussion

HFMD has become a serious public health problem in the Asia–Pacific region due to the large number of severe and fatal cases in a short period, and has been listed as a mandatory notifiable infectious disease in mainland China in 2008 [14, 15]. Our study showed that the majority of severe and dead HFMD cases occurred in children under 3 years of age, which was consistent with the previous studies in Guangxi and other provinces in China [15–17]. Most severe HFMD cases occur in children under 3-year-old, possibly due to the poor immune system of the young children. There was a higher prevalence among males than in females, which may be related to boys’ frequent exposure to enterovirus-contaminated environments or toys, where poor hygiene may increase the chance of infection [18, 19]. However, our study showed that there was no significant difference in clinical outcomes among different gender groups (Table 1), suggesting that gender had little effect on the disease progression.

In this study, we found that children who lived in the rural areas were more likely to develop severe HFMD, which is in line with previous studies in Guangxi [20]. Previous studies have found that poor medical conditions and poor guardian awareness of HFMD treatment are the influencing factors for the development of severe HFMD and mortality in rural areas in China [21–23]. Meanwhile, rural patients are more willing to seek medical treatment nearby, which is a possible reason for the higher mortality of HFMD in rural areas. Rural village or township health service centers often do not have adequate conditions for HFMD diagnosis and treatment. Because some HFMD cases have hidden or asymptomatic symptoms in the early stage, the inadequate capacity of village or township health service centers for diagnosis and treatment may lead to prolonged and inappropriate treatment. In addition, due to the limitations of medical resources and economic conditions, the mortality of HFMD is high in some regions in China, especially in the underdeveloped Guangxi [24, 25]. Given the fact that HFMD cases in Guangxi are most concentrated in rural areas, primary medical centers, including village and township health service centers, need to improve the ability of precision diagnosis and treatment, such as introducing new diagnostic technologies or instruments to make rapid and accurate diagnosis, so that HFMD patients can receive timely treatment.

Our study showed that severe HFMD without skin rash symptoms was a risk factor for death, which is consistent with other studies [26]. Skin rash is an obvious physical sign that may prompt the guardians to take their children to see the doctor, so the patients with skin rash may have more chances to meet the doctor and get timely treatment. On the other hand, HMFD patients without skin rash or other clinical symptoms may miss opportunity to visit the hospital because the guardians were unaware of the occurrence of HMFD, thus increasing the risk of developing severe HFMD and even death. Our study also found that the time interval from the first visit to diagnosis of severe HMFD was a significant risk factor for mortality. In general, a shorter time from the first visit to diagnosis should improve clinical outcomes. However, our results show an opposite trend (Table 2). We speculated that this may be related to insufficient awareness of HFMD among guardians, which often leads to delay in visiting to hospital. The delayed visit of hospital means that some patients will quickly become serious or critical in a shorter time, and these patients are most likely to die of cardiopulmonary failure due to delayed treatment. Therefore, in this sense, it is of particular importance to strengthen the education of guardians on HFMD knowledge and improve their awareness of children seeking medical treatment, which may become an effective strategy to prevent the death of HFMD in rural areas.

It is worth mentioning that an important finding of our study is the protective effect of EV-A71vaccination against death of severe HFMD cases. EV-A71 is the primary pathogen of HFMD and is also the main cause of severe and fatal HFMD cases [27, 28]. Previous studies have shown that inactivated monovalent EV-71 vaccine has a protective effect of about 85.4% against EV-71 virus infection [29], however, which also means that some immunized children will still infected with EV-71 virus. This raises the interesting question of whether there is a reduction in mortality in patients who remain infected with EVs after EV-A71 immunization. Our study provides a positive answer that EV-A71vaccination is effective in preventing death of severe HFMD cases, evidenced by the fact that EV-A71 vaccination protected 70.80% of the death of severe HFMD cases (Table 3). The protective effect of EV-A71 vaccination on mortality is of great significance, which is comparable with that of COVID-19 vaccination, although the vaccination has little protective effect on viral infection, it can greatly reduce the mortality [7, 30–33]. In addition, another interesting question is whether EV-71 vaccination protects against death from severe HFMD cases caused by other EVs infections, such as Cox-16. Our data showed that among 27 severe HFMD cases caused by Cox-16 infection, there were 3 deaths in EV-71 unvaccinated group but zero death in EV-71vaccinated group, preliminarily suggesting that EV-71 vaccination may have a protective effect against death caused by Cox-16 infection. Our current data is too few to do statistical analysis, but this is a direction worth focusing on in the future.

Our study has several advantages. First, this is a multicenter study, with samples from almost all medical institutions in Guangxi, covering almost all of severe HFMD cases in Guangxi during 2014–2018. Second, this study is a hospital-based study. Most of the data came from medical records, which are accurate and reliable. Third, the sample size is relatively large, facilitating statistical analysis. Meanwhile, this study has several limitations. First, the subjects enrolled in this study were hospitalized patients with severe HFMD. Because HFMD is a self-limited disease, not all the severe HFMD cases were hospitalized. Especially in village or township health service centers, a small number of patients were treated by combining outpatient and home medication. Second, the retrospective nature makes it less accurate in assessing the effect of vaccination than prospective clinical trials.

## Conclusions

Through a hospital-based epidemiologic study, we found that the mortality risk of severe HFMD in Guangxi was related to HFMD history of playmates, hospital grade, EV-A71 vaccination, correct diagnosis at first visit, Time interval from first visit to diagnosis of severe HFMD and rash symptom. More importantly, we found EV-A71 vaccination can significantly reduce mortality among severe HFMD cases. Given the fact that the HFMD epidemic in Guangxi is still serious and the coverage of EV-A71 vaccination is not very high, our findings are of great significance for the effective prevention and control of HFMD in Guangxi, and one of the priorities is to greatly improve the coverage of EV-A71 vaccination.

## Supplementary Information

**Additional file 1.** Comparison of demographic data of patients between vaccinated and not vaccinated group.

**Additional file 2.** Case Questionnaire for severe or fatal Hand, foot, and mouth Disease.

## Data Availability

The datasets used and/or analyzed during the current study are available from the corresponding author on reasonable request.

## Abbreviations

HFMD: Hand, foot and mouth disease
EV-A71: Enterovirus A 71
