# Supplementary material for: Factors related to the mortality risk of severe hand, foot, and mouth diseases (HFMD): a 5-year hospital-based survey in Guangxi, Southern China
Source: BMC Infect Dis. 2023 Mar 8;23:144. doi: 10.1186/s12879-023-08109-y (PMC9993373; doi:10.1186/s12879-023-08109-y)
Supplement: Supplementary file 1 — Additional file 1. Comparison of demographic data of patients between vaccinated and not vaccinated group. [file 12879_2023_8109_MOESM1_ESM.docx]

**Supplement 1 Comparison of demographic data of patients between vaccinated and not vaccinated group.**

| Characteristics | sum | vaccinated  (n=628) | not vaccinated  (n=925) | χ^2^ value | *P* value |  |
| --- | --- | --- | --- | --- | --- | --- |
| Gender |  |  |  |  |  |  |
| male | 989 | 411 | 578 | 1.42 | 0.23 |  |
| female | 564 | 217 | 347 |  |  |  |
| Age(years) |  |  |  |  |  |  |
| 0-1years | 155 | 66 | 89 | 5.66 | 0.13 |  |
| 1-2years | 684 | 302 | 382 |  |  |  |
| 2-3years | 411 | 157 | 254 |  |  |  |
| 3 years or older | 239 | 89 | 150 |  |  |  |
| Area | |  |  |  |  |  |
| urban | 270 | 120 | 150 | 2.32 | 0.13 |  |
| rural | 1278 | 504 | 774 |  |  |  |
| Registered residence | |  |  |  |  |  |
| long-term | 1518 | 612 | 906 | 0.01 | 0.91 |  |
| migrant | 29 | 12 | 17 |  |  |  |
| Group classification | |  |  |  |  |  |
| Kindergarten children | 2014 | 75 | 129 |  |  |  |
| scattered children | 1330 | 545 | 785 | 2.22 | 0.53 |  |
| School student | 16 | 7 | 9 |  |  |  |
| No. of Children 0 - 5 years old in the family | | | |  |  |  |
| ≤1 | 672 | 261 | 411 | 17.28 | <0.01 |  |
| 2-3 | 553 | 250 | 303 |  |  |  |
| ≥4 | 124 | 72 | 52 |  |  |  |
| Milk feeding way | |  |  |  |  |  |
| breast milk | 1150 | 1 090 | 61 | 22.83 | <0.01 |  |
| milk powder | 84 | 81 | 3 |  |  |  |
| mix feeding | 296 | 281 | 15 |  |  |  |
| Visiting hospital previously | | | |  |  |  |
| yes | 38 | 21 | 17 | 3.59 | 0.06 |  |
| no | 1503 | 601 | 902 |  |  |  |
| HFMD history of playmate in last 3 months | | | |  |  |  |
| yes | 228 | 84 | 144 | 1.37 | 0.24 |  |
| no | 1311 | 537 | 774 |  |  |  |
| History of chicken pox, eczema, etc. in last month | | | | |  |  |
| yes | 115 | 46 | 69 | 0.01 | 0.93 |  |
| no | 1424 | 576 | 848 |  |  |  |
| Time interval from disease onset to first visit to hospital | | | | |  |  |
| <1 day | 1310 | 535 | 775 | 4.59 | 0.10 |  |
| 1-2 days | 135 | 57 | 77 |  |  |  |
| ≥3 days | 102 | 31 | 71 |  |  |  |
| Hospital level of first visit | |  |  |  |  |  |
| village | 426 | 206 | 220 | 21.66 | <0.01 |  |
| township | 209 | 89 | 120 |  |  |  |
| county | 569 | 193 | 376 |  |  |  |
| city | 339 | 134 | 205 |  |  |  |
| Correct diagnose at first visit | | |  |  |  |  |
| yes | 1058 | 413 | 646 | 2.22 | 0.14 |  |
| no | 486 | 209 | 277 |  |  |  |
| Time interval from first visit to diagnosis of severe HFMD | | | | |  |  |
| ≤1 day | 735 | 305 | 430 | 2.25 | 0.33 |  |
| 1-2 days | 358 | 151 | 207 |  |  |  |
| ≥3 days | 449 | 169 | 280 |  |  |  |
| Severe diagnosis hospital level | | | |  |  |  |
| below county-level | 603 | 303 | 300 | 41.97 | <0.01 |  |
| above city level | 940 | 321 | 619 |  |  |  |
| Time from first visit to admission | | |  |  |  |  |
| ≤1day | 877 | 374 | 503 | 7.57 | 0.02 |  |
| 1-2day | 325 | 135 | 190 |  |  |  |
| ≥3day | 332 | 113 | 219 |  |  |  |
| Clinical severity at admission | | |  |  |  |  |
| mild | 308 | 142 | 166 | 6.75 | 0.03 |  |
| severe | 1133 | 449 | 684 |  |  |  |
| critical | 92 | 30 | 62 |  |  |  |
| Fever |  |  |  |  |  |  |
| yes | 1504 | 611 | 893 | 0.25 | 0.62 |  |
| no | 46 | 17 | 29 |  |  |  |
| Rash |  |  |  |  |  |  |
| yes | 1507 | 612 | 895 | 0.65 | 0.42 |  |
| no | 30 | 10 | 20 |  |  |  |
