# Supplementary material for: Factors related to the mortality risk of severe hand, foot, and mouth diseases (HFMD): a 5-year hospital-based survey in Guangxi, Southern China
Source: BMC Infect Dis. 2023 Mar 8;23:144. doi: 10.1186/s12879-023-08109-y (PMC9993373; doi:10.1186/s12879-023-08109-y)
Supplement: Supplementary file 2 — Additional file 2. Case Questionnaire for severe or fatal Hand, foot, and mouth Disease. [file 12879_2023_8109_MOESM2_ESM.doc]

**Case Questionnaire for severe or fatal Hand, Food, and Mouth Disease**

Categories：① Severe ②Death

Cases Number：

**一、General feature of the patients and family**

1.Patient Name：

2.Gender：①Male ②Female

3.Date of birth: Year Month Day

4. Age: years old months

5.Height: cm, Weight: kg

6.Classification of school age：

①Diaspora children ②nursery children

③students ④other

7.Address: City County (District) Township Village

8.Duration of residence： years months

9.Type of registered permanent residence：

①Permanent population (Residence or residence duration ≥6 months);

②Floating population (Residence time less than 6 months)

10.Registered residence：①Rural ② Rural-urban ③ Urban area

11.Family numbers: Children under 14-year-old

12. Parents name: Phone number：

**二、Situation of Symptoms, Visit hospital and Treatment**

1. Date of onset： Year Month Day

2. Date of first visit： Year Month Day

2.1 Hospital:

2.2Hospital category for first HFMD diagnosis：

①At village level hospital ②At township level hospital

③At county level hospital ④Above city level

2.3 Diagnosed HFMD in first visit：0: No, 1: Yes

3. date of critical diagnosed： Year Month Day

3.1 hospital:

3.2 Hospital category for severe diagnosis:

①at village level hospital ②at township level hospital

③at county level hospital ④above city level

4. Whether visiting village level (individual) medical institutions for treatment：

0: No 1: Yes. (If “Yes”, Fill in options 4.1 to 4.9. If “No”, Skip to option 5)

4.1Date of visit： Year Month Day

4.2 Duration of therapy： days

4.3 Diagnosed HFMD：0: No, 1: Yes

4.4 Medication：0: No, 1: Yes

4.5 Medication ways：

①Oral administration ② Intramuscular injection

③ Intravenous drip ④ Anal administration

⑤ Other

4.6 Whether using antipyretic medication：

0:No 1:Yes，

Date： Year Month Day

Drug name:

4.7 whether using hormonal drugs such as dexamethasone：

0:No 1:Yes，

Date： Year Month Day

Drug name:

4.8 Whether using antibiotic medication：

0:No 1:Yes，

Date： Year Month Day

Drug name:

4.9 Whether using antiviral drugs：

0:No 1:Yes，

Date： Year Month Day

Drug name:

5. Whether visiting township level medical institutions for treatment：

0: No 1: Yes. (If “Yes”, Fill in options 5.1 to 5.9. If “No”, Skip to option 6)

5.1 Date of visit： Year Month Day

5.2 Duration of therapy： days

5.3 Diagnosed HFMD：0: No, 1: Yes

5.4 Medication：0: No, 1: Yes

5.5 Medication ways：

①Oral administration ② Intramuscular injection

③ Intravenous drip ④ Anal administration

⑤ Other

5.6 Whether using antipyretic medication：

0:No 1:Yes，

Date： Year Month Day

Drug name:

5.7 whether using hormonal drugs such as dexamethasone：

0:No 1:Yes，

Date： Year Month Day

Drug name:

5.8 Whether using antibiotic medication：

0:No 1:Yes，

Date： Year Month Day

Drug name:

5.9 Whether using antiviral drugs：

0:No 1:Yes，

Date： Year Month Day

Drug name:

6. Whether visiting county-level medical institutions for treatment：

0: No 1: Yes. (If “Yes”, Fill in options 6.1 to 6.9. If “No”, Skip to option 7)

6.1Date of visit： Year Month Day

6.2 Duration of therapy： days

6.3 Diagnosed HFMD：0: No, 1: Yes

6.4 Medication：0: No, 1: Yes

6.5 Medication ways：

①Oral administration ② Intramuscular injection

③ Intravenous drip ④ Anal administration

⑤ Other

6.6 Whether using antipyretic medication：

0:No 1:Yes，

Date： Year Month Day

Drug name:

6.7 whether using hormonal drugs such as dexamethasone：

0:No 1:Yes，

Date： Year Month Day

Drug name:

6.8 Whether using antibiotic medication：

0:No 1:Yes，

Date： Year Month Day

Drug name:

6.9 Whether using antiviral drugs：

0:No 1:Yes，

Date： Year Month Day

Drug name:

7. Whether visiting above city level medical institutions for treatment：

0: No 1: Yes. (If “Yes”, Fill in options 7.1 to 7.9. If “No”, Skip to option 8)

7.1 Date of visit： Year Month Day

7.2 Duration of therapy： Days

7.3 Diagnosed HFMD：0: No, 1: Yes

7.4 Medication：0: No, 1: Yes

7.5 Medication ways：

①Oral administration ② Intramuscular injection

③ Intravenous drip ④ Anal administration

⑤ Other

7.6 Whether using antipyretic medication：

0:No 1:Yes，

Date： Year Month Day

Drug name:

7.7 whether using hormonal drugs such as dexamethasone：

0:No 1:Yes，

Date： Year Month Day

Drug name:

7.8 Whether using antibiotic medication：

0:No 1:Yes，

Date： Year Month Day

Drug name:

7.9 Whether using antiviral drugs：

0:No 1:Yes，

Date： Year Month Day

Drug name:

8. Last admission date: Year Month Day

8.1 Type of hospital admitted：

①At village level hospital ②At township level hospital

③At county level hospital ④Above city level

8.2 Condition on admission： ①Critical ②Severe ③Mild

8.3 Date of transfer to critical condition after admission:

Year Month Day

8.4 Whether admitted to the ICU：

0: No 1: Yes. (If “Yes”, Fill in options 8.4. If “No”, Skip to option 8.5)

Date： Year Month Day

Duration in ICU： Days

8.5 whether Endotracheal intubation (mechanical ventilation) or not：

0: No 1: Yes. (If “Yes”, Fill in options 8.5. If “No”, Skip to option 8.6)

Date： Year Month Day

Duration of Endotracheal intubation： Days

8.6 Date of death：

0: No 1: Yes. (If “Yes”, Fill in options 8.6. If “No”, Skip)

Date： Year Month Day

Diagnosis of death：

Principal Diagnosis:

Secondary Diagnosis:

**三、Medical history and other relevant information**

1. Weight at birth （g）

2. Gestational age , (If gestational age is unknown, Preterm birth: 0 No 1 Yes)

Parities： ,Childbirth：① Cesarean section ② Natural delivery

2. Complications during delivery:

0: No, 1:Yes, (Note: ）

3. Feeding way ：①Break milk ②Mix feeding ③Milk powder ④Other

4. Whether have Congenital heart disease, congenital malformation and other congenital diseases:

0: No, 1: Yes，Name：

5. Whether have an immune deficiency disease：

0: No, 1: Yes，Name：

6. Whether have a history of drug or food allergies：

0: No, 1: Yes，Name：

7. Whether have the vaccination card (certificate)：0: No, 1: Yes

8. Have been vaccinated one month before onset of illness (if no vaccination card, ask parents)：

0: No, 1: Yes, 9: Unclearly (If “Yes”, Please Fill out the form below)

| Vaccine | Time of vaccination | Vaccine | Time of vaccination |
| --- | --- | --- | --- |
|  |  |  |  |
|  |  |  |  |
|  |  |  |  |

9. Whether had measles, chickenpox, flu, cold, rubella, mumps and other infectious diseases in the month before onset:

0: No, 1: Yes，Date： Year Month Day

Disease Name：

10. Whether had a Fever 3 months before the onset of the disease：

0: No, 1: Yes，(if “Yes”, fill the 10.1-10.2, “No” skip to option 11)

10.1 Whether using antipyretic drugs: 0 No 1 Yes

10.2 Whether using the following drugs (multiple options):

① Aminopyrine ② Anemone ③ Enadidine

④ Lepyrine ⑤ Hormone (dexamethasone, etc.)

11. Who take care of the children in the family：

①Parents ② Grandparents ③ Relatives ④ Nanny ⑤ Others

Length of education for carer: years

Education level of carer:

① Illiterate ② Primary ③ Middle school

④ High school/Technical Secondary school ⑤ Junior college or above)

12. Whether had a HFMD history of playmates in last 3 months:

0: No, 1: Yes，Number：

13. Whether been to the hospital in the last 7 days：

0: No, 1: Yes，

Date： Year Month Day

Department of treatment:

Reason：

**四、Sample collection and test results**

1. Whether collecting specimens：0: No 1: Yes.

2. Specimen types:

① Throat swab ② Stool ③ Anal Swab

④ Herpes fluid ⑤ Other

3. Test results：①EV71 positive ②CoxA16 positive ③ Other enteroviruses

4. Sample test record

| Number of specimen | Specimen types | Date of sampling | Date of testing | Result | | | |
| --- | --- | --- | --- | --- | --- | --- | --- |
|  |  |  |  | Nucleic acid testing (NAT） | | Virus isolation | |
|  |  |  |  | RT-PCR | Realtime RT-PCR | RD | Hep-2 |
|  |  |  |  |  |  |  |  |
|  |  |  |  |  |  |  |  |
|  |  |  |  |  |  |  |  |
|  |  |  |  |  |  |  |  |

5. Specimen types and test results of all family members living with the child

| Number of specimen | name | gender | age | Relationship | have HFMD |
| --- | --- | --- | --- | --- | --- |
|  |  |  |  |  |  |
|  |  |  |  |  |  |
|  |  |  |  |  |  |
|  |  |  |  |  |  |

* Relationship with children :① parents ② grandparents ③ brothers and sisters ④ uncle/aunt ⑤ others (fill in the specific relationship).

|  |  |  |  | nucleic acid testing (NAT） | | virus isolation | |
| --- | --- | --- | --- | --- | --- | --- | --- |
|  |  |  |  | nucleic acid testing (NAT） | | virus isolation | |
| date of HFMD | Specimen types | Date of sampling | date of testing | RT-PCR | Realtime RT-PCR | RD | HEp-2 |
|  |  |  |  |  |  |  |  |
|  |  |  |  |  |  |  |  |
|  |  |  |  |  |  |  |  |

**五、Clinical symptoms and signs**

1. Fever：0: No 1:Yes，

Date： Year Month Day, time of duration： Days

2. Temperature for the first time： ℃；

Highest body temperature prior to visit： ℃;

Highest body temperature after admission： ℃

3. Rash：

0: No, 1: Yes，

Date： Year Month Day, Time of duration： Days

Type of rash：① Macular rash ② Papule ③ Blister rash ④ Others

Rash parts: ① Hand ② Foot ③ Mouth ④ Hip

⑤ Limbs ⑥ Torso ⑦ Other

4. Cough：0: No, 1: Yes

5. Other Symptoms：

6．Various Complications and/or Signs and date of appearance

| Complications and/or Signs | Date | Prior to admission | | | | At admission | |  | |  | |  |  |  |
| --- | --- | --- | --- | --- | --- | --- | --- | --- | --- | --- | --- | --- | --- | --- |
|  | Time |  |  |  |  |  |  |  | |  | |  |  |  |
| Nervous system | | | | | | | | | | | | | | |
| Headache | |  | | | |  | |  | |  | |  |  |  |
| Poor spirit | |  | | | |  | |  | |  | |  |  |  |
| Easily frightened | |  | | | |  | |  | |  | |  |  |  |
| Be on edge | |  | | | |  | |  | |  | |  |  |  |
| Tic | |  | | | |  | |  | |  | |  |  |  |
| Frequently convulsions | |  | | | |  | |  | |  | |  |  |  |
| Eclampsia | |  | | | |  | |  | |  | |  |  |  |
| Spasm | |  | | | |  | |  | |  | |  |  |  |
| Tremble | |  | | | |  | |  | |  | |  |  |  |
| Weakness of limb | |  | | | |  | |  | |  | |  |  |  |
| Acroparalysis | |  | | | |  | |  | |  | |  |  |  |
| Nuchal rigidity | |  | | | |  | |  | |  | |  |  |  |
| Kerning symptoms positive | |  | | | |  | |  | |  | |  |  |  |
| Weakened tendon reflexes | |  | | | |  | |  | |  | |  |  |  |
| Disappearance of tendon reflexes | |  | | | |  | |  | |  | |  |  |  |
| Somnolence | |  | | | |  | |  | |  | |  |  |  |
| Lethargy | |  | | | |  | |  | |  | |  |  |  |
| Light coma | |  | | | |  | |  | |  | |  |  |  |
| Deep coma | |  | | | |  | |  | |  | |  |  |  |
| Pupillary | |  | | | |  | |  | |  | |  |  |  |
| Pupillary light reflex | |  | | | |  | |  | |  | |  |  |  |
| Respiratory system | | | | | | | | | | | | | | |
| Cough | |  | | | |  | |  | |  | |  |  |  |
| Sore throat | |  | | | |  | |  | |  | |  |  |  |
| Nasal congestion | |  | | | |  | |  | |  | |  |  |  |
| Runny nose | |  | | | |  | |  | |  | |  |  |  |
| Tachypnea | |  | | | |  | |  | |  | |  |  |  |
| Bradypnea | |  | | | |  | |  | |  | |  |  |  |
| Dyspnea | |  | | | |  | |  | |  | |  |  |  |
| Changes in respiratory rhythm | |  | | | |  | |  | |  | |  |  |  |
| Die blausucht | |  | | | |  | |  | |  | |  |  |  |
| Foam (sputum) (0:no,1: white, 2: pink, 3: blood) | |  | | | |  | |  | |  | |  |  |  |
| Phlegmatic murmur | |  |  |  |  |  | |  | |  | |  |  |  |
| Moist rale | |  | | | |  | |  | |  | |  |  |  |
| Circulatory system | Date | Prior to admission | | | | At admission | |  | |  | |  |  |  |
|  | Time |  |  |  |  |  |  |  | |  | |  |  |  |
| Abnormal skin color | |  | | | |  |  | |  | |  | |  |  |
| Cyanosis of the fingers, toes, or lips | |  | | | |  | |  | |  | |  |  |  |
| Face, hands, feet  Pale and gray | |  | | | |  | |  | |  | |  |  |  |
| The body become cyanosis, pale, gray | |  | | | |  | |  | |  | |  |  |  |
| Skin pattern | |  | | | |  | |  | |  | |  |  |  |
| Tachycardia; (heart rate>120) | |  | | | |  | |  | |  | |  |  |  |
| Changes in heart rhythm (arrhythmia) | |  | | | |  | |  | |  | |  |  |  |
| Shallow pulse | |  | | | |  | |  | |  | |  |  |  |
| Weak pulse | |  | | | |  | |  | |  | |  |  |  |
| Cold limbs | |  | | | |  | |  | |  | |  |  |  |
| Digestive system | | | | | | | | | | | | | | |
| Vomiting | |  | | | |  | |  | |  | |  |  |  |
| Brown vomit | |  | | | |  | |  | |  | |  |  |  |
| Ventosity | |  | | | |  | |  | |  | |  |  |  |
| Diarrhea | |  | | | |  | |  | |  | |  |  |  |
| Melanemesis | |  | | | |  | |  | |  | |  |  |  |
| Hematochezia | |  | | | |  | |  | |  | |  |  |  |

(Note: Except for the following symptoms or signs, fill in the information as "0: No,1: Yes".

Pupil state: 1: normal, equal large and circle, 2 shrink, 3 scatter; Pupillary light reflex: 0 normal 1 abnormal; Decreased tendon reflexes: 0: No, 1: unilateral, 2: bilateral; Tendon reflexes disappeared: 0: No, 1: unilateral, 2: bilateral)

Investigator 1 _ __, Investigator 2

Institution ,Date： Year Month Day
